# Reconfigurable Tri‐Mode Metasurface for Broadband Low Observation, Wide‐Range Tracing, and Backscatter Communication

**DOI:** 10.1002/advs.202304879

**Published:** 2024-02-11

**Authors:** Jing Ning, Yilin Zheng, Shaojie Wang, Tian Jiang, Junming Zhao, Ke Chen, Yijun Feng

**Affiliations:** ^1^ Department of Electronic Engineering School of Electronic Science and Engineering Nanjing University Nanjing 210023 China

**Keywords:** low observation, reconfigurable metasurface, retro‐reflection, wide range tracing, wireless communication

## Abstract

In the current prevalent complex electromagnetic (EM) environment, intelligent methods for versatile and integrated control of EM waves using compact devices are both essential and challenging. These varied wave control objectives can at times conflict with one another, such as the need for broad absorption to remain inconspicuous, while also requiring enhanced backward scattering for highly reliable tracing and secure communication. To address these sophisticated challenges, a microwave‐frequency reconfigurable tri‐mode metasurface (RTMM) is introduced. The proposed innovation enables three distinct operational modes: broadband low observation, enhanced EM wave tracing, and backscatter communication over a wide‐angle range by simple control of the PIN diodes embedded in each meta‐atom. The proof‐of‐concept demonstration of the fabricated prototype verified the switchable tri‐mode performance of the RTMM. This proposed RTMM can be adapted to various applications, including EM shielding, target detection, and secure communication in complex and threatening EM environments, paving the way for environmentally‐adaptive EM wave manipulation.

## Graphical abstract

A multifunctional metasurface is presented that enables dynamically switchable electromagnetic (EM) scattering properties with real‐time control. It allows three distinct operation modes: broadband low‐radar observation, wide‐range tracing, and backscatter communication across a wide angular direction range. Consequently, the proposed metasurface may be adapted to various applications in complex and threatening EM environments, including EM shielding, target detection, and secure communication.

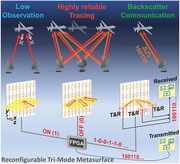

## 1 Introduction

The flexible manipulation of electromagnetic (EM) waves has long been a prominent objective in various fields of modern science and technology. This includes EM scattering control,^[^ ^1^, ^2^ ^]^ EM energy absorption,^[^ ^3^, ^4^ ^]^ wireless communication,^[^ ^5^ ^]^ and more. In practical applications, diverse wave functionalities are often simultaneously required for different technological aspects. For example, in the field of electronic warfare, the rapid advancement of radar technology has considerably increased the probability of detecting objects. This, in turn, has created a pressing need for low‐observable techniques to substantially minimize the EM scattering of an object. Conversely, there is a requirement for techniques that enhance EM backscattering enhancement to strengthen echo signals for highly‐reliable target tracking. This is crucial in numerous applications, including collision avoidance systems, life‐saving measures, and target detection.^[^ ^6^, ^7^ ^]^ Another example would be unmanned aerial vehicles (UAVs), which may require the ability of high trackability under particular EM waves to ensure long‐distance self‐aviation and wireless communication. However, they also need wideband and wide‐range low observability to avoid detection by adversarial EM wave detection.^[^ ^8^ ^]^ To meet these contradictory technological requirements, there is a high demand for reconfigurable EM devices that can integrate the diverse functionalities of EM wave manipulation. These devices enable the dynamic switching between different functionalities, allowing multiple tasks to be performed effectively.

Metasurfaces, which represent a 2D form of metamaterials, are composed of subwavelength meta‐atoms arranged on an ultrathin surface. They have demonstrated excellent ability in arbitrarily manipulating the amplitude, phase, and polarization of EM waves in both transmission and reflection configurations.^[^ ^9^, ^10^, ^11^ ^]^ Owing to their advantages of being low‐profile, lightweight, and easy to fabricate, numerous new devices have been developed, such as invisibility cloaks,^[^ ^12^, ^13^ ^]^ flat lenses,^[^ ^14^, ^15^ ^]^ imaging systems,^[^ ^16^, ^17^ ^]^ and more.^[^ ^18^, ^19^ ^]^ As a result, metasurfaces have become a promising platform for various EM devices and applications. In recent developments, conventional structure‐alone or passive metasurfaces have progressed toward reconfigurable and tunable metasurfaces. These metasurfaces are dynamically driven by external stimuli, such as thermal effects,^[^ ^20^, ^21^ ^]^ electrical tuning,^[^ ^22^, ^23^ ^]^ or mechanical stretching^[^ ^24^, ^25^ ^]^ to produce changeable EM functionalities. Compared to passive metasurfaces, which generally exhibit simple and static EM functionality, reconfigurable metasurfaces provide a more flexible means of regulating EM waves through time‐division multiplexing technology. This adaptability holds significant potential for practical applications across numerous fields.

Metasurfaces have found extensive use in achieving low EM observability through methods like EM wave absorption^[^ ^16^, ^26^, ^27^ ^]^ or diffusive scattering.^[^ ^28^, ^29^, ^30^ ^]^ These techniques are helpful for reducing radar cross section (RCS) and can also be adapted into a reconfigurable configurations by incorporating actively tunbale components. In contrast, designs that require high trackability often depend on a strong backward RCS, and may rely on retro‐reflection^[^ ^31^, ^32^ ^]^ or the integration of amplification elements in the wave‐reflection process.^[^ ^33^, ^34^ ^]^ However, most of these designs are limited in that they only function effectively at fixed angles,^[^ ^35^, ^36^, ^37^ ^]^ or require specific pre‐conditions pertaining to the incident angle to generate the desired phase gradient,^[^ ^38^, ^39^ ^]^ which significantly hinders their practical applications. Conversely, the Van Atta array,^[^ ^40^, ^41^ ^]^ a simple and common retro‐reflection method, comprises passive or active antenna elements connected in pairs by transmission lines. It is capable of providing a phase distribution of reradiated fields that is the reverse of that of the received fields, thus, reradiating the wave back toward the incident direction, achieving retro‐reflection at arbitrary incident angles. Despite the prevalence of these functional metasurfaces, it remains a challenge to integrate both low observability and wide‐angle high scattering, which appear to contradict each other, as well as other advanced wave functionalities.

**Figure 1:**
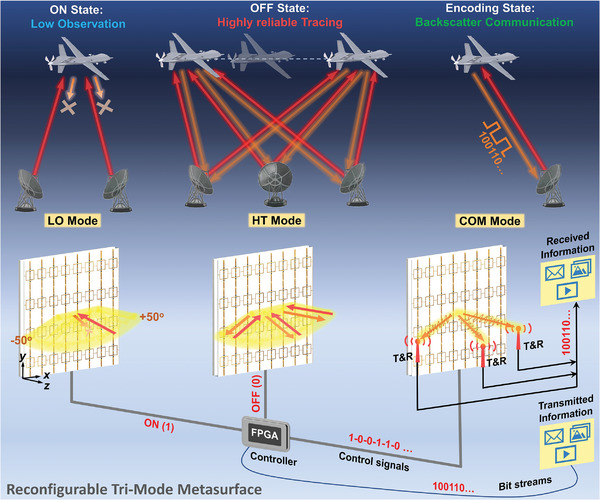
The conceptual illustration of the reconfigurable tri‐mode metasurface and its potential application in UAV flight missions. In LO‐mode (left panel), all the PIN diodes loaded on the metasurface are switched to ON state, enabling wideband absorption with wide angular stability. In highly reliable tracing (HT) mode (middle panel), the PIN diodes are all switched to the OFF state, allowing the metasurface to achieve wide‐angle retro‐reflection (or Van Atta property). In COM‐mode (right panel), the PIN diodes are dynamically switched between ON and OFF states, and the control‐voltage sequences are applied from the field‐programmable gate array (FPGA) to the PIN diodes on the metasurface for transmitting information through the physical channel.

To address these conflicting requirements, we propose a reconfigurable tri‐mode metasurface (RTMM) capable of dynamically switching its EM scattering properties with real‐time control. This RTMM combines the Van Atta array structure with a reconfigurable metastructure, offering retro‐reflection performance (or Van Atta property) as well as to attain multifunctional EM wave controls. The RTMM can operate in three distinct working modes: broadband low observation, wide‐range tracing, and backscatter communication, as schematically illustrated in **Figure** **1**. The concept of backscatter communication has a historical precedent dating back to the infamous Great Seal Bug.^[^ ^42^ ^]^ It involves modulating an antenna's impedance to encode information into already existing waves, effectively overcoming the challenge of limited spectral resources and energy consumption of conventional wireless communication architectures.^[^ ^43^ ^]^ In the low‐observation mode (LO‐mode), the metasurface achieves a significant reduction in broadband scattering over a wide range of angles through EM wave absorption. Meanwhile, in the retro‐reflection mode, the metasurface realizes retro‐reflection that considerably enhances backscattering over a wide range of incident angles. Moreover, by time modulation of the above two functions, digital information can be directly encoded onto the reflected wave. This approach allows for realizing highly directive and secure signal transmission, making it valuable for applications in wireless communication and radar systems. Leveraging a highly directional scattering beam that carries modulation information facilitates the realization of secure communication^[^ ^44^ ^]^ and a more elaborate physical‐layer scheme can be achieved based on perfect absorption conditions.^[^ ^45^ ^]^ Here, the proposed communication mode (COM‐mode) achieves a confidential and secure mode of information transmission since the transmitted information can only be received in the direction of the receiving terminal. This is because the RTMM scatters the information‐carrying reflection wave along the incident direction with high directionality and forms a relatively narrow beam (beam width about 10°). Therefore, the position different from the receiver (outside the beam direction) is typically incapable of receiving the backscattered wave energy and cannot eavesdrop the transmitted information, ensuring a secure wireless communication. Target tracking can be realized, as the position of the receiver changes without the need for additional beam modulation. As a result, the information channel always follows the receiver position with high directivity, ensuring a confidential and secure mode of communication. Moreover, the retro‐reflection scheme employed in the COM‐mode exhibits well‐focused and directionally‐modulated backscattering, making it less susceptible to the effects of random rich scattering or multipath scattering. Furthermore, this property can be extended complex EM environments with rich scattering.^[^ ^46^ ^]^ Metasurface‐based backscatter communication schemes can efficiently transmit information to a receiver by actively modulating the backscatter through a space‐coding sequence.^[^ ^44^, ^45^ ^]^ In contrast, the proposed COM‐mode utilizes the retro‐reflection beam and has a unique property in which the receiver can be located at an arbitrary position within a wide angular range, without requiring additional beam modulation on the metasurface side. All of these modes can be switched in real time using a shared aperture regulated by an external controller to fulfill the requirements of information transmission in complex environments, such as UAV flight missions. In the following sections, we present the design principles of the RTMM, as well as simulation and measurement results that validate the reliability and functionality of the proposed tri‐mode metasurface.

## 2 Concept and Results

To attain the contradictory functionalities of the RTMM, the proposed metasurface must have the capability to simultaneously control the reflection amplitude and phase. This control is necessary to manipulate both the dissipation and direction of the reflected wave, respectively. In this study, the RTMM employs a composite strategy in its design, involving two distinct metastructures: 1) Switchable resistive layer (SRL): A top‐layer metastructure utilized to switch between EM wave absorption and transmission, and 2) Retro‐reflection layer (RL): A bottom‐layer metastructure employed to control the reflection phases. The upper SRL operates in the resistive and transparent states to dissipate or transmit incident waves, respectively, by controlling the microwave PIN diode in each unit cell. The bottom RL is designed as a Van Atta array that can modulate the reflection phases of the waves transmitted from the SRL. It achieves this by flipping the phase gradient along the center of the structure under arbitrary incident angles. By executing this phase modulation, the RL creates a phase distribution in the radiated field that is the reverse of that in the received field, enabling the metasurface to retro‐reflect incoming EM waves. Additionally, alternative EM waves retro‐reflection structures may be employed in the reconfigurable metasurface to achieve the conflicting functionalities of the RTMM. Here, the Van Atta structure is just one of the viable implementation approaches.

On this basis, the RTMM seamlessly integrates two key functionalities: wide‐band absorption for LO‐mode and wide‐angle retro‐reflection for highly reliable tracing (HT‐mode). The transition between these modes can be accomplished in real‐time by simply changing the bias voltages on the PIN diodes loaded within the SRL. In the LO‐mode, all PIN diodes are biased with ON states, and the proposed RTMM can achieve broadband EM wave absorption with good angular stability. Conversely, in the HT‐mode, all PIN diodes are switched to the OFF state, causing the SRL to become transparent. In this state, the RTMM structure achieves retro‐reflection through the RL at its central working frequency. This leads to a noticeable enhancement in the backward radar cross‐section (RCS) over a wide range of incident angles. Notably, the proposed RTMM operates under *y*‐polarization excitation.

Moreover, the dynamic switching between the two modes provides a remarkable contrast in the magnitudes of the reflected waves in the incident direction. This contrast can serve as binary states, representing “0” for low reflection and “1” for high reflection. Consequently, a wireless information communication mode can be established, based on the binary amplitude‐shift keying (BASK) modulation of the backscattered wave. This modulation is achieved by encoding the metasurface in real‐time. In the COM‐mode, the PIN diodes operate in an “Encoding” state, which is dynamically switched between ON and OFF states using a field‐programmable gate array (FPGA) controller. This switching action allows for real‐time modulation of the amplitude of the reflected waves. In this manner, a metasurface‐assisted wireless communication scheme, employing BASK‐modulation, can be constructed. Importantly, this approach eliminates the need for complex frequency‐mixing and radio‐frequency modules. Instead, it directly modulates the carrier waves through the metasurface to encode the digital information.^[^ ^44^, ^45^ ^]^

**Figure 2:**
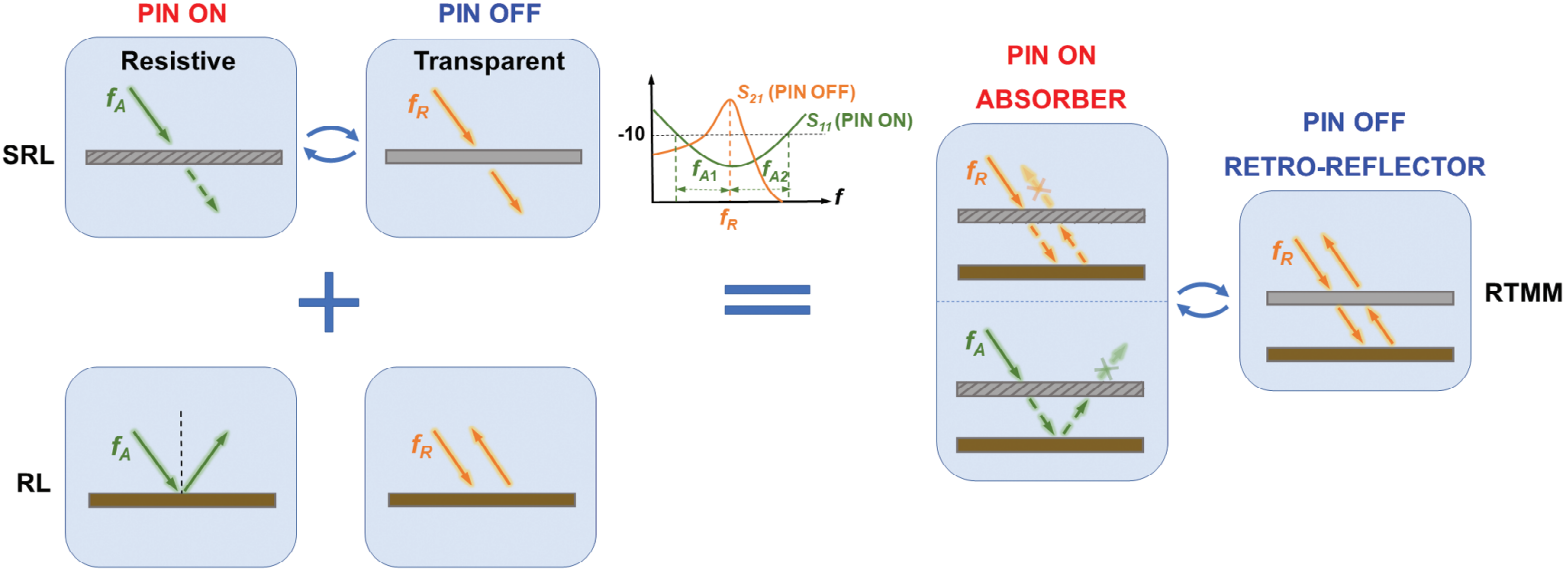
Principle illustration of the proposed RTMM. The working states of the PIN diodes are dynamically controlled by the bias voltage from the external FPGA controller.

Next, we delve into the detailed design requirements of the RTMM, considering two critical aspects: SRL at the top and the RL at the bottom. The working principle of the composite strategy is illustrated in **Figure** **2**. The SRL transition between a resistive and a transparent sheet is based on the ON to OFF states of the loaded PIN diodes, respectively. In the resistive state, it dissipates the incident wave across a broad absorption frequency range (denoted as *f_A_*_1_ and *f_A_*_2_), whereas in the transparent state, it allows the incident wave to pass through at the frequency of *f_R_*. The RL is designed to retro‐reflect the illuminated EM waves at a frequency of *f_R_* and to retain specular reflection at other frequencies. Thus, the composite structure can transition between absorber and retro‐reflector modes by electrically switching the PIN diodes. Furthermore, the performance of absorption and transparency in the RL must be stable at oblique incidences, ensuring wave absorption and retro‐reflection with a broad angular range. For the RL design, the working frequency band should align well with the passband of the SRL to achieve retro‐reflection of EM waves with minimal loss.

### 2.1 Design of the SRL Meta‐Atom

**Figure 3:**
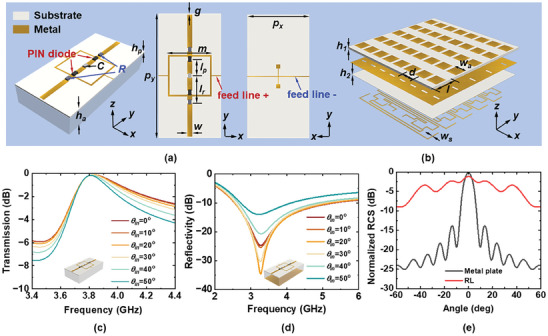
Schematic of the proposed RTMM and the simulation results. a) The schematic, top, and backward view of the SRL. b) The schematic of the RL. c) The simulated transmission performance of the SRL meta‐atom under different incident angles when all loaded PIN diodes are in the OFF state. d) The simulated absorption performance of the SRL meta‐atom under different incident angles when all loaded PIN diodes are in the ON state. The inset illustrates that a metal ground is added when achieving the absorption ability, which is replaced by the RL in the composite structure. e) The simulated monostatic RCS of the designed RL compared to that of the metal plate with equal size.

**Figure** **3**a depicts the structure of the proposed SRL, which is composed of two short wires connected with a thin metallic square ring. These two wires were coupled with a capacitor (denoted as *C*) in the center, each of which was serially loaded with a PIN diode and a resistance (denoted as *R*). Copper metallic wires and rings with a thickness of 0.018 mm were printed on an F4B dielectric substrate with a relative permittivity of 4.4, a loss tangent of 0.001, and a thickness of *h_p_* = 0.3 mm. The lumped elements loaded in the meta‐atom were a chip capacitor with capacitance *C* = 2 pF, and two lumped resistors with resistance *R* = 200 Ω. Two PIN diodes were used to separate the central wire from the resistor‐loaded wires to control the operating state of the SRL. A thin metal square ring, connected to the positive pole of the PIN diodes, was also utilized to provide a direct current (DC) bias voltage for switching the diodes between the ON and OFF states. The negative pole was connected to the bias line at the back of the substrate through a metal via. The geometric parameters of the meta‐atom were optimized as *p_x_* = 15 mm, *p_y_* = 30 mm, *m* = 10.5 mm, *l_p_* = 4 mm, *l_r_* = 6.3 mm, *g* = 0.3 mm, and *w* = 0.6 mm. When all the diodes are switched to the OFF state, the SRL operates in a parallel resonance to realize an infinite impedance.^[^ ^47^ ^]^ Thus, a lossless transmission can be achieved at the resonance frequency of *f_R_*. When the loaded PIN diodes are switched to the ON state, the SRL performs as dispersive coupled wires that can be represented as a resistive sheet. By cascading a metal ground plane with an air spacer of thickness *h_p_* = 10 mm to the SRL, broadband absorption (with reflectivity less than −10 dB) is realized similarly to that of a circuit analog absorber (CAA).^[^ ^48^, ^49^, ^50^ ^]^ The working mechanism can also be analyzed using the surface current distributions, as shown in Section S1 and Figure S1 (Supporting Information).

To validate the EM response of the proposed SRL meta‐atom, a full‐wave EM simulation was conducted using commercial software. The meta‐atom was illuminated using a *y*‐polarized EM wave, with periodic boundary conditions along the lateral sides, and Floquet ports along the longitudinal sides. The simulation setup considers the mutual coupling between the meta‐atoms, assuming that all meta‐atoms have uniform states. The PIN diode was modeled as a resistor‐inductor‐capacitor (RLC) series circuit with parameters of *R_p_* = 0.5 Ω and *L_p_* = 0.7 nH for the ON state, and *L_p_* = 0.5 nH and *C_p_* = 0.24 pF for the OFF state. The transmission and absorption performances of the meta‐atoms are shown in Figure 3c,d. Notably, the simulated absorption represents the SRL meta‐atom when cascaded with a metal ground, as analyzed above. This metal ground can be replaced by RL in the realized integrated structure, as detailed in Section S3 (Supporting Information). It can be observed that when the PIN diodes are turned off, the meta‐atom provides a well‐defined passband with a central frequency of ≈3.85 GHz. The insertion loss (IL) remains below 0.5 dB even as the incident angle increases up to 50°. Conversely, when the diodes are switched to the ON state, the absorption bandwidth ranges from 2.55 to 5.31 GHz with good angular stability. These simulation results verified that the proposed SRL satisfied the design requirements mentioned above and could manipulate EM waves by simply switching the PIN diodes in real‐time.

### 2.2 Design of the RL

The proposed RL located at the bottom of the RTMM is schematically illustrated in Figure 3b. It comprises three metallic layers separated by two dielectric substrates, functioning as a conventional Van Atta array. It is composed of five linear subarrays along *x*‐axis, each containing four aperture‐coupled square‐patch antenna pairs. On the backside of the RL, two patch antennas in pairs are connected by equal‐length microstrip lines, allowing them to function as both receivers and reradiators, ensuring retro‐reflection functionality. The distance between the subarrays (denoted by *l*) and the period of the patch elements (denoted by *d*) were set to 48 and 30 mm, respectively. The patch antenna used had a side length of *w_a_* = 23.8 mm, resonating at an operating frequency of 3.85 GHz. An aperture‐coupled configuration was employed, and a slot with dimensions of 3.5 × 13.8 mm^2^ was cut into the middle metallic ground layer. The width of the microstrip feed line was *w_s_* = 2.1 mm. The two dielectric substrates supporting the metallic patterns were made of F4B. The first substrate had a relative permittivity of 2.2, a loss tangent of 0.001, and a thickness of *h_1_* = 2 mm. The second substrate had a relative permittivity of 3.5, a loss tangent of 0.001, and a thickness of *h_2_* = 0.45 mm. The structure parameters were optimized to ensure that the antenna element had a 50 Ω input impedance (as seen by the feed line) and a return loss of −19.5 dB at the center frequency of 3.85 GHz. This design guarantees that the antenna pairs can receive and reradiate the maximum power, which is analyzed in Section S2 (Supporting Information). Simulations were conducted of the far‐field performance of the proposed RL under *y*‐polarized plane waves at different incident angles. Figure 3e plots the normalized monostatic RCS of the RL compared with that of an equal‐sized metal plate at 3.85 GHz. The findings reveal a clear RCS enhancement by the RL compared to the equal‐sized metal plate under different oblique incidences. Notably, the RL can accurately retro‐reflect the incident wave back toward the incident direction, even at incident angles up to ±50°, achieving backward RCS enhancement over a wide angle range. The retro‐reflected beam had a return loss of ≈1 dB at normal incidence. This loss can be primarily attributed to dielectric loss and imperfect matching between the feed lines and antennas.^[^ ^41^ ^]^ These results were also supported by simulated 2D scattering patterns under *y*‐polarized illumination at different incident angles, as plotted in Figure S3 (Supporting Information).

### 2.3 Tri‐Mode Metasurface

**Figure 4:**
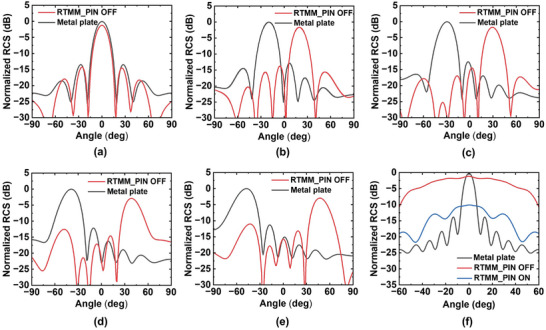
The simulated retro‐reflection performance of the proposed RTMM under *y*‐polarized illumination at an operating frequency of 3.85 GHz. The simulated 2D scattering patterns of the RTMM compared to that of a same‐sized metal plate at different incident angles of a) 0°, b) 20°, c) 30°, d) 40°, and (e) 50°. f) The simulated monostatic RCS of the RTMM with PIN diodes in ON and OFF states.

The RTMM can be created by cascading the designed SRL and RL together with a 10 mm air space. Within the RTMM, the RL serves a dual role, acting as a metallic ground with out‐of‐phase total reflection in the absorption band and functioning as a retro‐reflector at the transparent frequency of the SRL, set at 3.85 GHz. By replacing the metallic ground in Figure 3d with RL, a similar broadband absorption characteristic is maintained in the composite RTMM, as all the PIN diodes are switched to the ON state. This is demonstrated in Figure S4 (Supporting Information), where the amplitudes of the scattering beam in  all backward directions were kept below −10 dB, showcasing a broad bandwidth (3–5 GHz) and a wide angular range (0° ± 50°). Next, when the PIN diodes are switched to the OFF state, the retro‐reflection performance of the RTMM is calculated at its central working frequency of 3.85 GHz, aligning with the passband frequency of the SRL and the retro‐reflection frequency of the RL, as shown in **Figure** **4**. The simulated 2D scattering patterns under different incident angles compared to that of the same‐sized metal plate are plotted in Figures 4a–e, indicating the efficient retro‐reflection ability of the RTMM that can accurately reradiate the incident wave along the incident direction within the broad angle range of 0±50°. The retro‐reflected beam exhibited a return loss of ≈1.2 dB when incident at normal angles, which increased gradually as the incident angle deviated from normal. This is because the retro‐reflection efficiency of the RTMM is affected by the deterioration of the reception and coupling performances of the meta‐atom as the incidence angle increases. Additionally, we simulated the monostatic RCS of the metasurface when all PIN diodes were switched to the ON or OFF state. The results were compared with that of a metal plate of the same size, as illustrated in Figure 4f. It is evident that a significant enhancement in backward RCS can be observed when the diodes are turned off, accompanied by a notable difference in reflection amplitudes across a wide range of incident directions as the diodes transition between the OFF and ON states. For a more detailed explanation of how the scattering beam is manipulated as the PIN diodes change state, the simulated far‐field scatterings of the RTMM under different states of the PIN diodes at the retro‐reflection frequency of 3.85 GHz are available in Section S3 (Supporting Information).

### 2.4 Operation Principle of the Backscatter Communication

As shown in Figure 1, the proposed RTMM also realizes backscatter communication. The information (for example, text, pictures, or videos) is first transformed into a binary bit stream. This stream is then synchronously encoded onto the reflections of the incident waves by the RTMM. The encoding process is achieved by switching the PIN diodes using the bias voltage signal output from a computer‐controlled FPGA. Finally, the modulated carry wave signals are retro‐reflected, and the information can be received and interpreted by the receiver terminal.

In this metasurface‐assisted direct wireless communication, information encoding, and signal modulation are realized directly using a metasurface as the modulator. Similar to the previously proposed metasurface‐assisted wireless communication system,^[^ ^51^ ^]^ for the BASK‐modulation scheme we chose, a mapping relationship between the reflection coefficient *Γ_m_* (*m* = 0 or 1 for “ON” or “OFF” state, respectively) and the digital codes in a BASK system can be established as:

(1) $$\Gamma_{0}=A_{0}e^{j\varphi }\Leftrightarrow^{\prime }0^{\prime },\Gamma_{1}=A_{1}e^{j\varphi }\Leftrightarrow^{\prime }1^{\prime }$$

Where *A*_0_ and *A*_1_ are the different reflection amplitudes of the metasurface at the “ON” and “OFF” state respectively, and *φ* is the reflection phase that is identical for different discrete states. This relationship indicates that the switchable reflection coefficient of the proposed RTMM with distinct amplitude differences can be defined as 1‐bit binary digits “0” and “1,” supporting the backscatter communication with BASK‐modulation.

## 3 Experimental Verifications

**Figure 5:**
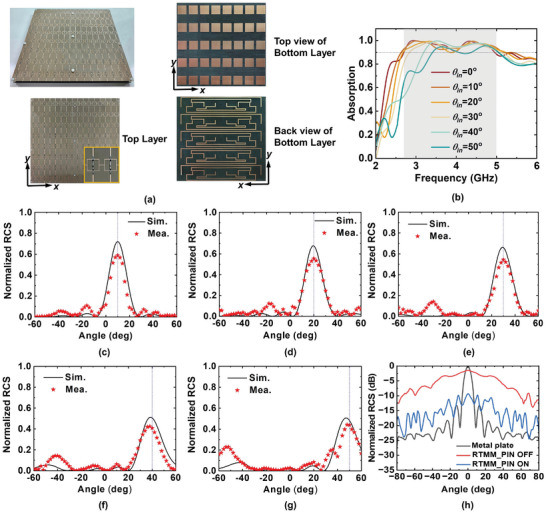
Photographs of the fabricated RTMM and measured performance of absorption and retro‐reflection under ON and OFF states of PIN diodes with *y*‐polarized illumination. a) Photographs of the RTMM prototype. b) Measured absorption performance of the RTMM under different incident angles with all loaded diodes turned to ON state. The comparison of measured and simulated results of 2D scattering patterns with loaded diodes switched to OFF under various incident angles of c) 10°, d) 20°, e) 30°, f) 40° and g) 50°. The measured retro‐reflection results are normalized to the specular reflection power of a same‐sized metallic plate under the same incident conditions. h) The measured monostatic RCS of the RTMM with PIN diodes in ON and OFF states, compared with that of the metal plate with equal size.

To experimentally validate the design and functionalities of the RTMM, we fabricated a prototype using printed circuit board (PCB) technology and conducted scattering measurements and backscatter communication. The SRL (containing 16 × 8 meta‐atoms) and RL, both measuring 240 mm × 240 mm, were cascaded together with an air spacer using plastic screws, as illustrated in **Figure** **5**a. The sample was illuminated by *y*‐polarized waves, with the incident interface defined as the *xoz* plane. To verify the EM wave absorption performance of the RTMM, we measured its reflectivity in a standard microwave chamber with two linearly polarized broadband horn antennas serving as the transmitter and receiver. Horn antennas can be freely moved along a mechanical arc to measure the angular performance. More details on the testing setup can be found in Section S4 (Supporting Information). The measured results for different incident angles with the loaded PIN diodes in the ON state are plotted in Figure S6b (Supporting Information), showcasing a low reflectivity of less than −10 dB for specular reflection under different incident angles. To further illustrate that the incident energy dissipated rather than scattered in other directions, we measured the scattering patterns of the RTMM when the PIN diodes were switched to the ON state. The measurement setup and results are described in Section S4 (Supporting Information) and illustrated in Figures S7 and S8 (Supporting Information). The amplitude of the scattering beam is basically maintained below −10 dB in all directions, ensuring a good energy absorption feature. The absorption curves were obtained by subtracting the integrated scattering energy in all directions from the incident energy (Figure 5b). The results indicate that the metasurface can attain more than 90% absorption in the bandwidth from 2.7 to 5 GHz under normal incidence. This corresponds to a 59.7% fractional bandwidth, with good angular stability at the central frequency. However, the absorbing performance at low frequencies deteriorates slightly with an increase in the incident angle.

Next, we switched the loaded PIN diodes to the OFF state, measured the 2D scattering patterns of the RTMM at various incident angles, and compared them with the simulated results to verify their retro‐reflection functionality (as shown in Figures 5c–g). Although a subtle frequency shift was observed in the measurements, particularly in terms of the best retro‐reflection operating frequency (3.83 GHz), the measured reflection beams remained aligned along the incident direction as the incident angle increased to 50°, which agrees well with the simulations. The measured retro‐reflected energy was slightly lower than the simulated energy, likely due to potential losses incurred during the packaging and welding of the lumped elements, as well as the imperfections in fabricating and measuring the sample. Figure 5h illustrates the measured monostatic RCS of the RTMM, with PIN diodes switching between the ON and OFF states at 3.83 GHz. The measurement system is described in Section S4 and Figure S9 (Supporting Information). When the loaded PIN diodes change to the OFF state, the RTMM exhibited a significant RCS enhancement (≈15 dB) compared with the metal plate under oblique incidence. According to the radar equation,^[^ ^52^ ^]^ the maximum radar detection distance for an object is proportional to *σ* ^1/4^, where *σ* is the RCS of the object. Therefore, the RTMM, operating in the HT mode, can extend the radar detection range by ≈2.4 times compared to the original value. In addition, there was a noticeable amplitude difference (exceeding 5 dB) between the two states within the incident angle ranging from −80° to 80°. This amplitude difference is more than sufficient to support the RTMM‐assisted backscatter communication system. Considering the fabrication and assembly tolerances, these measured results validate the design methodology and predicted functionalities of the RTMM.

**Figure 6:**
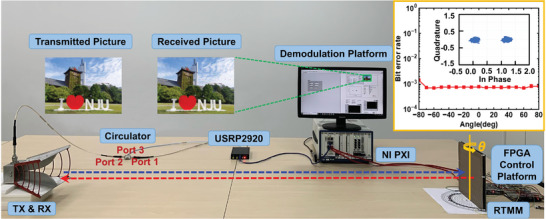
The constructed RTMM‐assisted wireless communication system. The insets show the variation trend of the bit error rate with the change of incident angle *θ* and the measured constellation diagram of the wireless communication system.

To validate the RTMM‐assisted backscatter communication, a realistic wireless communication system was established to perform a direct data transmission experiment in an indoor scenario, as shown in **Figure** **6**. The test system comprised a transmitter that was mainly composed of the FPGA control platform, the fabricated RTMM, and a receiving terminal containing a commercial demodulation platform (the universal software radio peripheral (USRP) 2920 and NI control platform PXIe‐1092, National Instruments Corp.). The linearly polarized horn antenna transmits carrier waves as the transmitter, receives modulated signals as the receiving terminal, and is connected to a circulator for transceiver integration. The baseband signal generated by the USRP is first transmitted to port 1 of the circulator and then illuminates the RTMM through the horn antenna at port 2. The modulated signal received by the horn antenna is then transmitted back to the USRP from ports 2 and 3 of the circulator. The separation of the transmitting and receiving signals of the transceiver antenna requires only the circulator, and no additional modifications to other hardware components of the communication system are required.

First, the transmitted information (for example, a picture) is translated into a sequence of binary symbols by sequentially replacing each pixel with 24‐bit binary data. The bit streams were then applied through the bias of the PIN diodes to drive the RTMM in real‐time through the FPGA control platform. Hence, the carrier waves illuminating the RTMM sample were directly modulated by time‐variant bit streams, which were then scattered and received by the transceiver antenna. Subsequently, the received signals are transmitted to the USRP to retrieve the carrying information. A threshold power value was set in advance to make threshold decisions for BASK‐modulated baseband signals. When the detected power is higher (lower) than the threshold value, the demodulator judges the received digital information as the binary symbol “1” (“0”). In the experiment, a color picture was used as an example and transformed into digital control signals to drive the RTMM. The carrier waves (baseband signals) illuminated on the RTMM at a frequency of 3.83 GHz illuminated on the RTMM were modulated and scattered in the incident direction and received by the transceiver antenna. The modulated baseband signals are then transmitted to the USRP and demodulated into binary symbols via a threshold decision. Finally, the demodulated signals retrieved the transmitted picture in real‐time via the NI control platform, as shown in the inset of Figure 6. Owing to the high directivity of the emitted signal from the antenna and the retro‐reflected signal from the RTMM, most of the energy is concentrated in the transmission channel without significant interference from the table or other items in the room.

To further prove the wide angular range of the backscatter communication, we also conduct an experimental test by rotating the RTMM sample along the central axis to achieve different incident/receiving angle of *θ*, mimicking cases for different receiver positions in a certain angular range. In the test, the picture can be fully recovered at the receiving terminal within a wide range of incident angle from −80° to 80°, and the bit error rate (BER) is basically below 1 × 10^−3^ by comparison with the original picture, as shown in the inset of Figure 6. The BER was calculated as the proportion of demodulated error symbols relative to the total number of symbols by comparing the received picture with the original picture. To verify the modulation scheme of the transmitter, a measured constellation diagram representing the 1‐bit binary symbols is provided in the inset of the BER graph, in which the distance of each point to the origin indicates the reflection amplitude of the binary symbol. Here, two constellation points are observed in the diagram, representing binary symbols “0” and “1,” thus verifying the BASK modulation. The two distinct constellation points observed in the demodulated signals indicate the high distinguishability of the two coding patterns and a relatively high SNR at the receiving terminal, which ensures a low BER for the received information.^[^ ^44^ ^]^ By analyzing the fluctuations of the received signal points on the constellation diagram, the SNR of the system can be estimated to be ≈13.4 dB; the methodology for this estimation is described in detail in Section S5 (Supporting Information). The transmission rate in the experimental test was ≈780 kbps, and further improvement in the transmission rate could be achieved by applying a well‐designed external biasing circuit and high‐speed data lines to precisely control the bias voltage. There are other options for switching meta‐atoms using high‐speed sensing devices and PIN diodes with higher switching speeds. The distortionless transmission distance reached 2 m with a transmit power of 1.6 mW in the experimental test and could be improved by increasing the transmit power. For practical applications, the transmission power of the transceiver can be increased significantly to the kilowatt level, which inevitably improves the communication range of the proposed backscatter communication system to accommodate long‐distance communication. Notably, compared with other metasurface‐assisted backscatter communication systems,^[^ ^53^, ^54^, ^55^ ^]^ the COM‐mode based on the proposed RTMM has the unique advantage of a wide angular range and high directionality of data transmission through retro‐reflection beams and implements a more flexible, highly efficient, secure, and energy‐saving backscatter communication method. The designed RTMM can realize retro‐reflection in a wide angular range from −80° to +80°. Therefore, by transmitting EM waves to the RTMM, the receiver at an arbitrary position within the wide range above can receive the retro‐reflected beam from the RTMM carrying the modulated information in real‐time, thereby achieving efficient backscatter communication. This particular backscatter communication method based on retro‐reflection waves provides a new approach to focus on user location, and can realize tracking as the position of the receiver changes. In principle, a metasurface can always scatter a narrow beam directly to the receiver without the need for additional beam regulation or sensor modules on the metasurface side, thereby simplifying the architecture and modulation process of communication systems. The communication channel always follows the receiver position with high directivity, and can therefore achieve a confidential and secure mode of communication. In addition, because the information transmitter (RTMM) does not need to emit microwave energy, the receiving terminal only consumes energy when receiving information, and the radiation beam from the RTMM has high directionality, the proposed COM‐mode also has the advantages of low energy consumption and low EM radiation pollution.

The RTMM prototype was successfully verified to achieve a switchable tri‐mode of broadband low observation, wide angular range tracing, and backscatter communication in 5G communication frequencies (n77 band) by the dynamic manipulation of the loaded PIN diode states. Although this study combines known functions on a metasurface, it overcomes the design obstacle of contradictory performance between different functions. Without losing generality, the proposed scheme and architecture can be scaled to other frequency bands and extended to a polarization‐independent operation, which may be realized by optimizing the meta‐atom structure of the SRL and adding feed apertures and networks of the RL.

## 4 Conclusion

In summary, we successfully proposed a reconfigurable tri‐mode metasurface that enables dynamically switchable EM scattering properties with real‐time control. This metasurface seemingly integrates contradictory EM functionalities, dynamically transitioning from wideband EM wave absorption to highly efficient retro‐reflection across a broad angular range by simply controlling the ON and OFF states of the PIN diodes embedded in each meta‐atom. Such multifunctional characteristics empower the proposed metasurface to operate in three distinct modes, suitable for broadband low‐radar observation, wide‐range tracing, and backscatter communication over a wide angular direction range. All of these functionalities were experimentally confirmed, demonstrating the excellent performance of the metasurface in diverse EM wave manipulations within a shared aperture. We also anticipate that the proposed reconfigurable metasurface platform has the potential for scalability to other frequency bands and can play a pivotal role in various applications that require highly reliable tracing and information transmission in complex threatening EM environments.

## Conflict of Interest

The authors declare no conflict of interest.

## Supporting information

Supporting Information

## Data Availability

The data that support the findings of this study are available from the corresponding author upon reasonable request.
