# Supplementary material for: Reconfigurable Tri‐Mode Metasurface for Broadband Low Observation, Wide‐Range Tracing, and Backscatter Communication
Source: Adv Sci (Weinh). 2024 Feb 11;11(15):2304879. doi: 10.1002/advs.202304879 (PMC11022728; doi:10.1002/advs.202304879)
Supplement: Supplementary file 1 — Supporting Information [file ADVS-11-2304879-s001.pdf]

ADVANCED SCIENCE
Open Access

Supporting Information

for *Adv. Sci.*, DOI 10.1002/advs.202304879

Reconfigurable Tri-Mode Metasurface for Broadband Low Observation, Wide-Range Tracing, and Backscatter Communication

Jing Ning, Yilin Zheng, Shaojie Wang, Tian Jiang, Junming Zhao, Ke Chen and Yijun Feng**

Supporting Information

Reconfigurable Tri-Mode Metasurface for Broadband Low Observation, Wide-range Tracing, and Backscatter Communication

Jing Ning, Yilin Zheng, Shaojie Wang, Tian Jiang, Junming Zhao, Ke Chen and Yijun Feng**

1. Analysis of surface current distributions on the SRL meta-atom

The operating mechanism of switching between absorption and transmission can also be explained by investigating the surface current distributions on the switchable resistive layer (SRL) meta-atom, as illustrated in **Figure S1**. At the central frequency of passband (i.e., 3.85 GHz), the surface currents are mainly distributed within the metal square ring when the PIN diodes are in OFF state, resulting in transmission with low loss. When the PIN diodes are in ON state, the currents pass through and are mostly disspated by the lumped resistors, resulting in effective EM energy absorption.

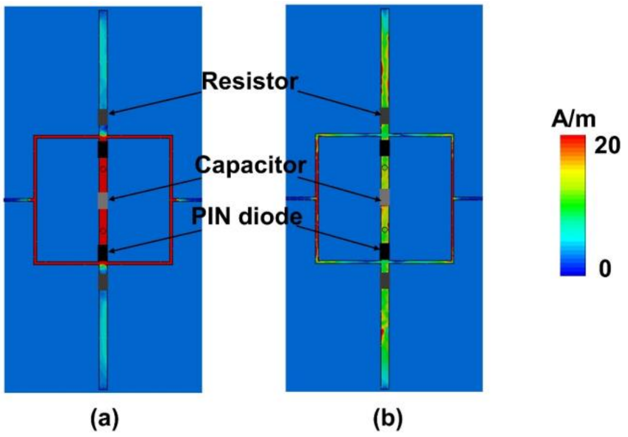

Figure S1. The surface current distributions on the SRL meta-atom at 3.85 GHz with PIN diodes of (a) OFF state and (b) ON state.

2. Simulated results of the RL

To design a well-matched retro-reflection layer (RL) for retro-reflection, the parameters of the antenna element are optimized to resonate at the required operating frequency. **Figure S2**a, b show the structure of the antenna element, the tuning stub length (l_s) of the feed line is 5.5 mm.

A discrete port is setted in the feed line terminal to monitor the return loss. The optimized element resonates at 3.85 GHz and has a return loss of -19.5 dB, which is plotted in Figure S2c. The simulated two-dimensional (2D) scattering patterns of the RL under different incident angles are shown in **Figure S3**, verifying that the RL can accurately retro-reflect the incident wave back towards the incident direction till the incident angle up to 50°.

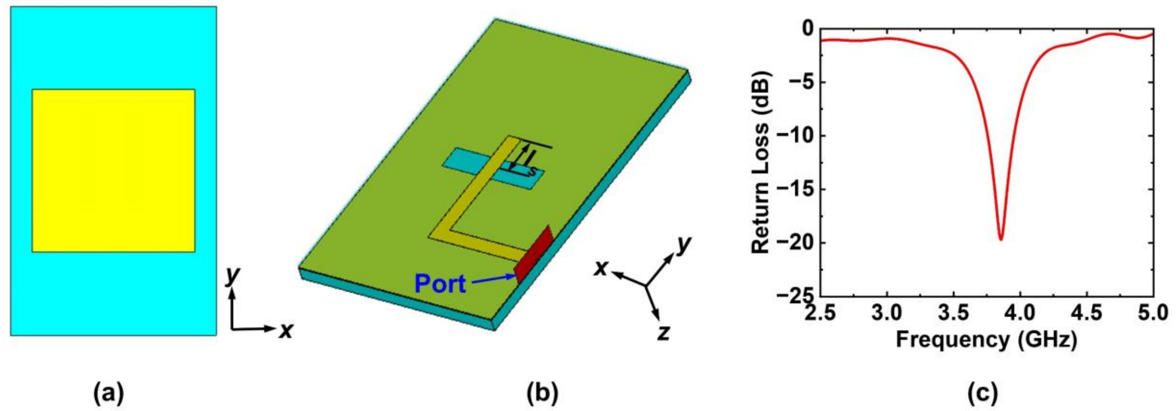

Figure S2. The antenna element and the simulated return loss of the RL. (a) The top view of the antenna element. (b) The back view of the antenna element. (c) The simulated return loss of the structure.

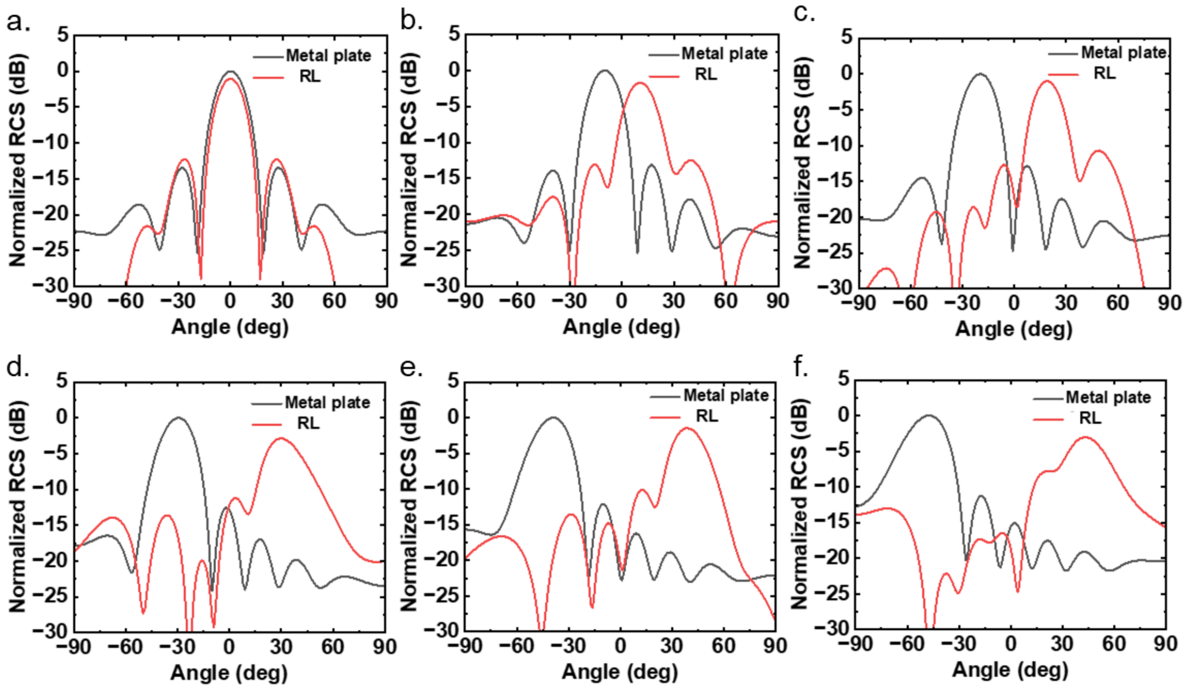

Figure S3. The simulated 2D scattering patterns of the RL compared with the equal-sized metal plate under *y*-polarized illumination at different incident angles of (a) 0°, (b) 10°, (c) 20°, (d) 30°, (e) 40°, and (f) 50°. The observatation frequency is set as 3.85 GHz.

3. Simulated results of the RTMM

We cascade the designed SRL and RL together with an air space to form the reconfigurable tri-mode metasurface (RTMM) and simulate the scattering patterns to verify the absorption performance when all PIN diodes are switched on. The results are shown in **Figure S4** and the incidence is set as *y*-polarized illumination with different incident angles. Within the absorption bandwidth of the SRL, the amplitude of the scattering beam is basically maintained below -10 dB in all directions, which is calibrated to the specular reflection of a same-sized metallic plate under same incidence. The results verify the absorption ability of the SRL when replacing the cascaded metal ground with the RL in the integrated structure. The three-dimensional (3D) far field scattering patterns of the RTMM at 3.85 GHz under different incident angles are plotted in **Figure S5**. The results in top and bottom row are the PIN diodes switched to OFF and ON states, respectively. The scattering waves are along the incident direction as loaded PIN diodes turned to OFF state, while most of the energy is absorbed when PIN diodes are switched to ON state.

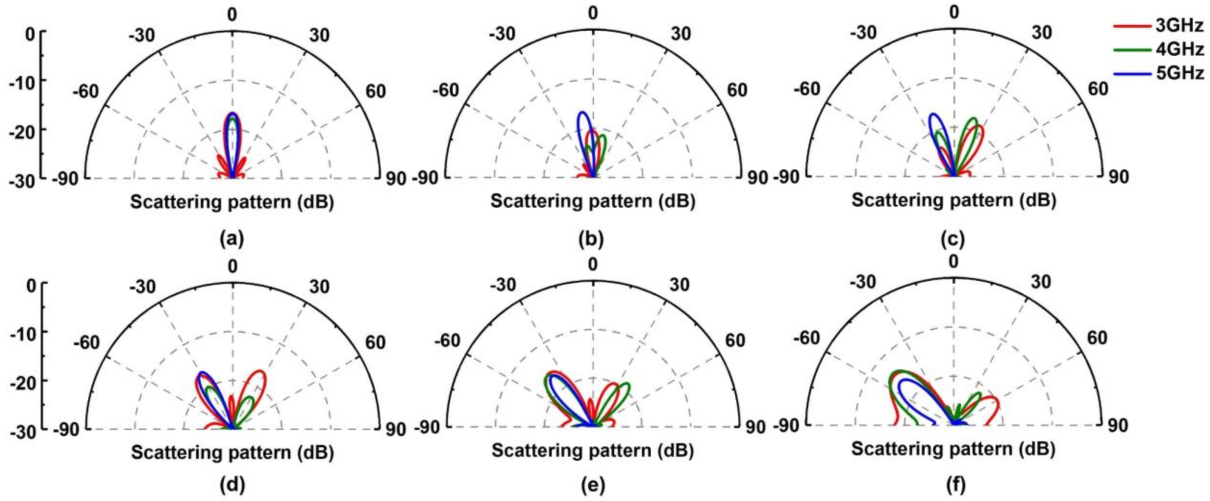

Figure S4. The simulated 2D scattering patterns of the RTMM under ON states of PIN diodes with *y*-polarized incidence. The incident angles in (a) - (f) are changing from 0° to 50° with the step of 10°, and the results are calibrated to the specular reflection amplitude of an same-sized metallic plate under same incident condition.

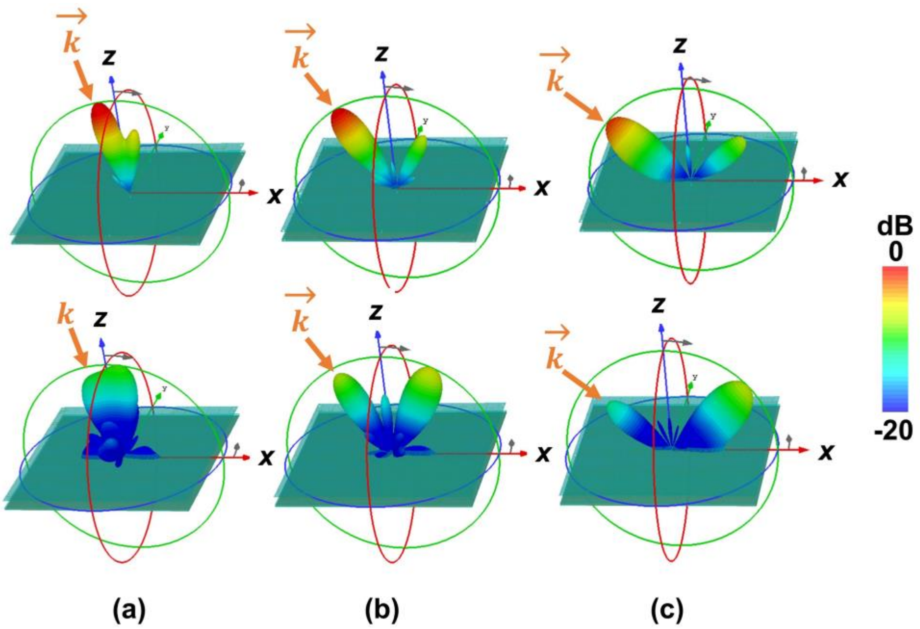

Figure S5. The simulated 3D far field scattering of the RTMM at 3.85 GHz with loaded PIN diodes switching between ON and OFF states. The results are under *y*-polarized illumination and incident angles are (a) -10°, (b) -30° and (c) -50°. The results in top and bottom row are the cases when the PIN diodes are switched to OFF and ON state, respectively.

4. Details of the measurement setups and results

A photograph of the test scenario for reflectivity measurement is shown in **Figure S6**a. The standard transmitting and receiving horn antennas are placed at equal distances from the sample to measure the reflectivity. Two linearly polarized broadband horn antennas are connected to the two ports of the vector network analyzer (Agilent N5244), and can be freely moved along a mechanical arc to measure reflectivity under different incident angles. The incident angles are changing from 0° to 50° with the step of 10°. The measured results under different incident angles with the loaded PIN diodes in ON state is plotted in Figure S6b, which is calibrated to the specular reflection of a same-sized metallic plate under same incidences. As the whole RTMM is a reflective slab, the low reflectivity less than -10 dB indicates that the metasurface can attain more than 90% absorption in the bandwidth from 2.7 to 5 GHz under normal incidence.

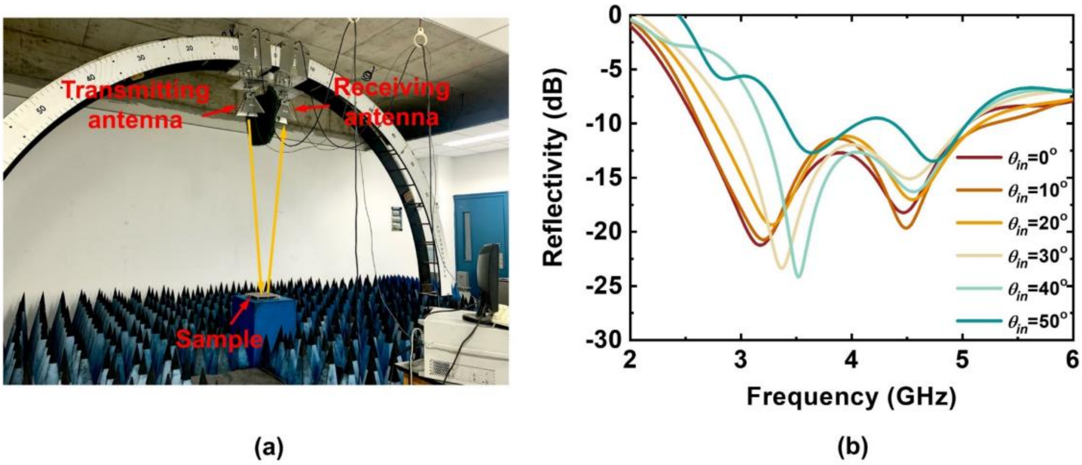

Figure S6. (a) Photograph of the test scenario for reflectivity measurement. (b) The measured reflectivity of the RTMM under different incident angles with loaded diodes turned to ON state.

A photograph of the test scenario for scattering patterns measurement is shown in **Figure S**7. The sample is placed in the center of an arc track, and a pair of linearly polarized horn antennas working as the transmitter and receiver are connected to the two ports of the vector network analyzer. One is mounted on the arc track serving as the transmitter source, while the other horn antenna works as the receiver standing behind the transmitting antenna to measure the scattered fields at various angles. The transmitting antenna and the sample are fixed on the turntable to rotate in the horizontal plane together, while the sample can rotate along the vertical axis for mimicking different incident angles. When the transmitting antenna together with sample move along the arc track, the scattered fields reflected by the sample can be recorded by the receiver. The two antennas will be inevitably overlapped in the incident direction because they are placed in the same horizontal plane. To solve this problem, the transmitting and receiving antennas are placed on the two sides of the surface normal of the sample with an equal pitch angle of 5° in the vertical plane. There is no phase gradient along the vertical direction on the sample, the oblique incidence will be reflected to specular direction in the vertical plane, which can be detected by the receiving antenna without any blockage in whatever directions.

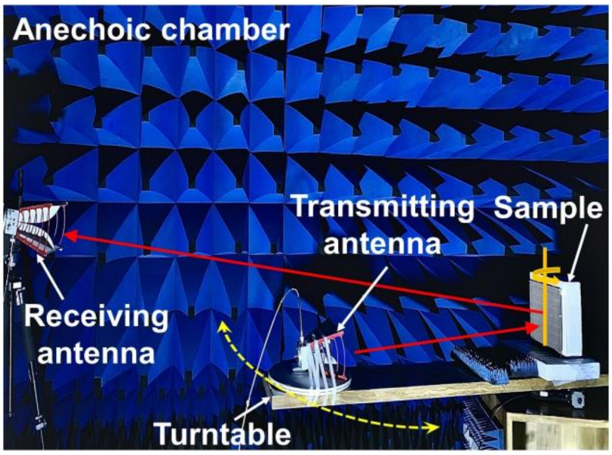

Figure S7. Photograph of the test scenario for scattering patterns measurement.

To further illustrated the incident energy is absorbed rather than scattered to other directions, we measure the scattering patterns of the RTMM when PIN diodes are switched to ON state, as shown in **Figure S8**. The measured results are consistent with the simulations, the calibrated amplitude of the scattering beam is basically maintained below -10 dB in all directions. The increased scattering amplitude under large-angle incidence indicates that the absorption performance in low frequency deteriorates as the increase of the incident angle,which is in line with the measurement results in Figure S6b.

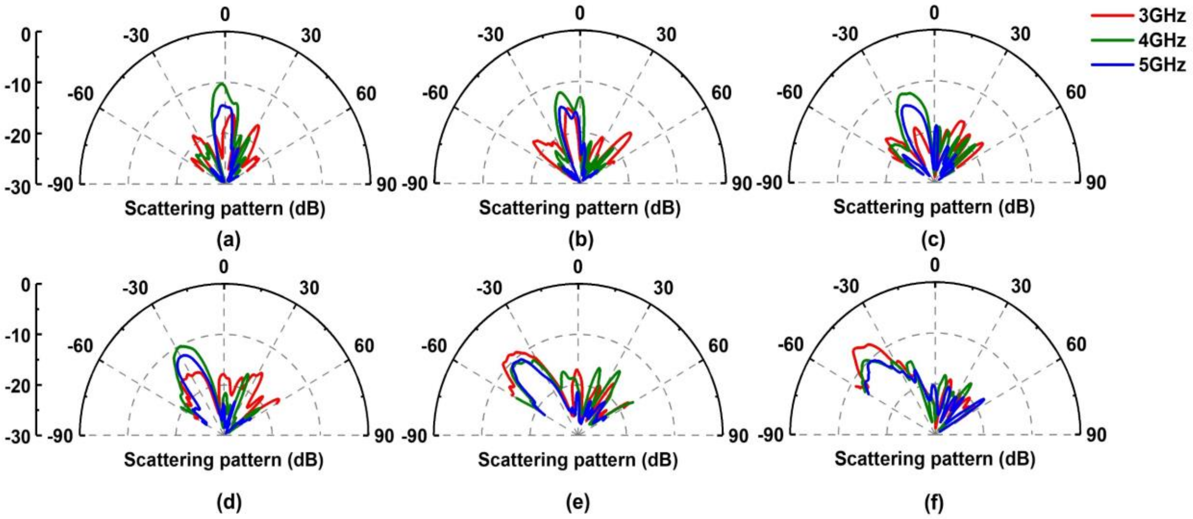

Figure S8. The measured 2D scattering patterns of the RTMM under ON state of PIN diodes with *y*-polarized illumination. The incident angles in (a) - (f) are changing from 0° to 50° with a step of 10°, and the results are calibrated to the specular reflection amplitude of a same-sized metallic plate under same incident condition.

A photograph of the experimental setup for the monostatic RCS measurement is shown in **Figure S9**. The sample is fixed on a turntable that could rotate 360° in the horizontal plane. Two standard linearly polarized horn antennas are connected to the two ports of a vector network analyzer and placed far enough from the sample: one serves as a transmitter and the other as a receiver, thus, it can be approximately considered that the two antennas are at the same position to realize the monostatic RCS measurements. During the measurement, the turntable rotates from -80° to 80° to obtain the RCS performance in this angle range.

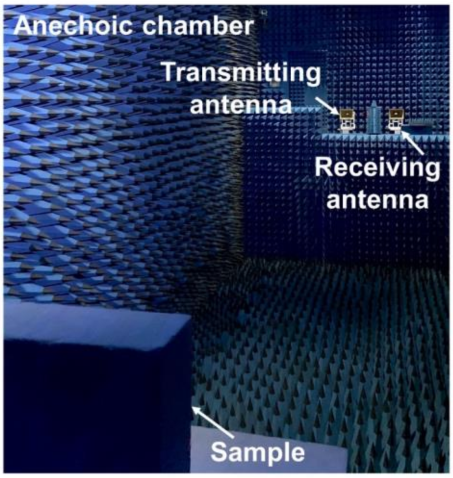

Figure S9. Photograph of the test scenario for the monostatic RCS measurement.

5. The estimated SNR for the communication system

The SNR of the wireless communication system can be estimated by performing numerous repetitions of the nominally identical measurement and observing its fluctuations. The measured constellation diagram, as depicted in **Figure S10**, illustrates the signal points received for numerous repeated measurements, and the marked red points represent the position of the ideal signal points. Using the measured constellation diagram, the error vector magnitude (EVM) can be calculated to evaluate the performance of a digital communication system. The equation utilized to calculate the EVM for each received signal point is as follows[1]:

$$\mathrm{EVM} = \sqrt{(I_{received} - I_{ideal})^2 + (Q_{received} - Q_{ideal})^2}\,, \quad (1)$$

where I and Q are the in-phase and quadrature signal amplitudes of the modulations. The EVM for the total system can be determined by the equation as:

$$\text{EVM\_system} = \left(\frac{average(EVM)}{A_{ideal}}\right) \times 100\%\,, \quad (2)$$

where A_{ideal} represents the ideal magnitude, which is the distance between the ideal signal point and the center point of the constellation diagram. Then, the SNR of the wireless communication system can be estimated using the relationship between EVM and SNR, as indicated below[1,2]:

$$\text{EVM\_system} = \frac{1}{\sqrt{SNR}}. \quad (3)$$

Therefore, the SNR of the communication system is approximately 13.4 dB, as estimated by the measured constellation diagram, which ensures a low BER for the received information.

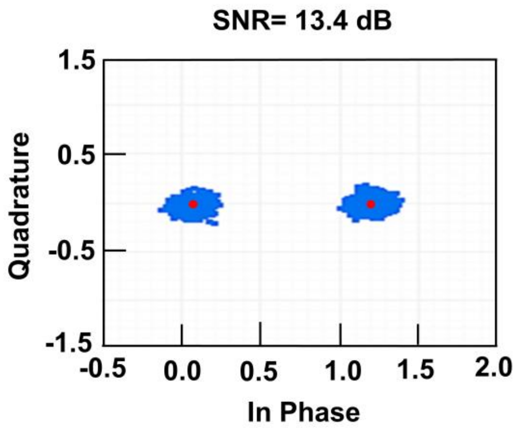

Figure S10. The measured constellation diagram and the estimated SNR of the wireless communication system.

References

[1] H. A. Mahmoud, H. Arslan, *IEEE Trans. Wirel. Commun.* **2009**, *8*, 2694.

[2] R. A. Shafik, M. S. Rahman, A. H. M. R. Islam, *Proc. 4th Int. Conf. Electr. Comput. Eng. ICECE*, **2006**, 408.
